# Exercise Interventions for Cognitive and Functional Outcomes in Dementia: A Systematic Review and Meta-Analysis Exploring Dose Metrics, Heterogeneity, and Implementation-Relevant Factors

**DOI:** 10.3390/healthcare14050689

**Published:** 2026-03-09

**Authors:** Chun-Wei Lu, Tsz-Ching Ng, Yi-Chen Cheng, Chun-Hsien Su

**Affiliations:** 1 Graduate Institute of Sport Coaching Science, Chinese Culture University, Taipei 111369, Taiwan; jimmy132457@gmail.com (C.-W.L.); chingng1106@gmail.com (T.-C.N.); 2 Department of Business Administration, College of Management, Shih Chien University, Taipei 104336, Taiwan; yc.elaine@g2.usc.edu.tw; 3 Department of Physical Education, Putian University, Putian 351100, China; 4 Department of Exercise and Health Promotion, Chinese Culture University, Taipei 111369, Taiwan

**Keywords:** dementia, healthy aging, structured exercise, cognitive outcomes, functional outcomes, adherence, meta-analysis, heterogeneity, meta-regression, implementation

## Abstract

**Background**: Exercise interventions are commonly considered as non-pharmacological approaches to support cognitive and functional outcomes in older adults with dementia. However, the effects reported in the literature remain heterogeneous, and commonly used time-based dose markers may be insufficient to explain variability across trials. **Methods**: A systematic review and meta-analysis of randomized controlled trials was conducted in accordance with PRISMA 2020 guidelines. Eligible trials described benefits with cognitive, functional or behavioral changes associated with structured exercise interventions in older adults with dementia. Random-effects meta-analysis and meta-regression models were used to derive pooled effects and assess if linear dose indicators (e.g., duration of intervention, session length, frequency and total cumulative dose) reflected heterogeneity. **Results**: Twenty-two studies were analyzed. Based on our pooled analyses, a small but statistically significant improvement was observed under the fixed-effects model (g = 0.106, 95% CI 0.015–0.197; *p* = 0.023), but this was not significant for random-effects models (g = 0.117, 95% CI −0.021–0.254; *p* = 0.097), while suggesting moderate between-study heterogeneity (Q(21) = 43.530, *p* = 0.003; I^2^ = 51.757%; τ^2^ = 0.052). For the main random-effects meta-regression, standard linear dose indicators did not significantly explain between-study heterogeneity (Qm(3) = 1.06, *p* = 0.7867; R^2^_analog ≈ 0), while significant residual heterogeneity remained (I^2^ ≈ 56.03%). **Conclusions**: In the literature so far, there are limited and heterogeneous effects of exercise interventions on cognition and functions in older adults with dementia. These findings in all literature suggest that the current evidence does not support a consistent linear dose–response relationship but rather will likely depend to some extent on feasibility and supervision (again, quality of the interventions), thus emphasizing that exercise strategies must be contextually sensitive rather than dose-dependent.

## 1. Introduction

Dementia is a progressive neurodegenerative disorder that causes disability and loss of independence in the elderly. The prevalence of dementia among individuals aged 65 years and older is estimated to range between approximately 5% and 8% globally, with projections suggesting a substantial increase in the number of affected individuals by 2050 as populations age [1,2]. Alzheimer’s disease (AD), the most common subtype, is characterized by gradual changes in the capacity to remember, retain executive function, and carry out activities of daily living on their own [3,4]. In addition to its impact on clinical care, dementia is a significant socioeconomic burden, with the global cost reaching over US$1 trillion every year, imposing chronic strain on health and long-term care systems [5]. The prevalence among patients aged ≥65 years was reported as 8.04% in Taiwan, highlighting the need for evidence-based initiatives to guide aging care, long-term services, and public health planning [6].

While pharmacological treatments primarily target neurotransmitter modulation and symptom management, non-pharmacological approaches such as structured exercise may influence broader neurobiological and systemic pathways, including neurotrophic signaling, vascular function, metabolic regulation, and functional reserve. These multidimensional mechanisms may contribute to maintaining cognitive and functional capacity through pathways not directly addressed by pharmacotherapy.

However, existing drugs (including cholinesterase inhibitors and N-methyl-D-aspartate (NMDA) receptor antagonists) have modest symptomatic benefits and do not significantly slow the progression of the disease [7,8], notwithstanding decades of pharmacological research. Recent disease-modifying strategies have been controversial to the extent that clinical relevance to disease outcomes, safety, and cost-effectiveness in practice care settings are debated [9,10]. Non-pharmacological interventions have been seen as being important means of preserving function, delaying decline, and increasing quality of life in older adults. This includes such interventions as systematic exercise, which is commonly recommended because the programs are highly scalable, relatively inexpensive, and target multiple pathways that are pertinent to cognitive and functional health in dementia care [11,12,13].

There are many neurobiological mechanisms through which exercise can influence cognition, including better cerebral perfusion, enhanced neurotrophin signaling, such as brain-derived neurotrophic factor (BDNF), and metabolic regulation, decreased neuroinflammation [14,15,16]. Animal studies demonstrate exercise-induced hippocampal neurogenesis and synaptic plasticity, and in addition, we report a healthy relationship between regular physical exercise and enhanced brain structure and functioning and executive function in humans [14,17,18]. Crucially, physical activity might also reduce behavioral and psychological symptoms of dementia (BPSD) like depressive symptomatology, agitation, and apathy, leading to lower levels of BPSD, and hence increase general well-being and functioning [11,19].

However, the clinical evidence is varied. Existing systematic reviews and meta-analyses have reported small to moderate benefits of exercise for dementia or cognitive impairment, but the size of effects in these studies differ significantly [11,12,13,19,20,21]. This variability is probably attributed not only to variations in exercise prescription variables (frequency, intensity, time, and type), but also heterogeneity in participant, intervention, compliance, outcome, and trial protocols [22,23,24,25,26,27]. However, from the healthcare and aging care context, this heterogeneity has critical implications for the feasibility and real-world exercise-moderation plans. Of great importance, though “exercise dose” is often mentioned as a significant source of variability, several previous syntheses have either taken the figure of the actual dose to be categorical or quantified dose–response relations using only continuous methods [22]. As a result, whether and how dose, particularly total weekly exercise volume, explains between-study variability to some extent remains poorly defined. However, prior syntheses have not consistently examined whether commonly reported exercise dose parameters explain between-study variability in outcomes.

The primary aim of this study was to examine whether commonly reported exercise dose parameters explain variability in cognitive and functional outcomes across randomized trials in dementia. Despite growing evidence from randomized trials and meta-analyses that structured exercise might have small, context-dependent, cognitive as well as functional benefits for individuals with dementia, there is major uncertainty about how exercise dose should be conceptualized and optimized for practical care. Exercise dose has conventionally been treated as a linear construct, such that greater volume or duration of prescribed workout correlates with greater benefits, a hypothesis which has been complicated by neurodegenerative limitations and frailty, and adherence challenges common in aging cohorts. As such, we performed a systematic review and meta-analysis of randomized controlled trials aimed specifically at quantitatively analyzing the impact of structured exercise interventions on older adults (≥65 years) living with dementia or cognitive impairment, reflecting the overlap in exercise trials conducted across these populations, with a focus on clarifying dose-related patterns relevant to both clinical and community-based care. We assessed several domains of outcomes, including cognitive function, functional ability, and mood or behavior, and evaluated exercise dose with prespecified continuous measures, including total weekly exercise volume (minutes/week), program duration (weeks), session duration (minutes/session), and accumulated exposure (total dose). Validity of trials was tested according to the Cochrane Risk of Bias 2 (RoB2) framework [28].

## 2. Materials and Methods

### 2.1. Protocol and Reporting

This systematic review and meta-analysis were conducted in accordance with the PRISMA 2020 statement and guidance for quantitative evidence synthesis. An a priori, prespecified analytic plan was implemented to (i) estimate pooled effects of structured exercise interventions in older adults with dementia or cognitive impairment and (ii) examine whether exercise dose parameters explain between-study heterogeneity using subgroup analyses and random-effects meta-regression [29]. The review protocol was prospectively registered in PROSPERO (CRD420251167113). Compliance with the PRISMA 2020 reporting guidelines is documented in the PRISMA 2020 checklist (Supplementary Table S1).

### 2.2. Eligibility Criteria

Studies were eligible if they met the following criteria:

Design. Randomized controlled trials (parallel-group RCTs).

Population. Older adults (≥65 years) with dementia (any subtype) or cognitive impairment. Inclusion of both dementia and cognitive impairment populations was permitted, given the overlap in exercise-based randomized trials across these groups; this approach reflects the pragmatic structure of the available evidence base.

Intervention. Structured exercise programs (e.g., aerobic, resistance, multicomponent, balance, dual-task, or exergaming) with sufficient reporting to derive dose parameters.

Comparator. Usual care, wait-list, non-exercise control, or attention control.

Outcomes. Quantitative outcomes falling into at least one prespecified domain—cognitive function (primary), functional ability, or mood/behavior—with adequate pre/post summary statistics (mean, SD, and sample size) to compute standardized effect sizes.

Studies were excluded if they were nonrandomized, lacked usable outcome data, or did not provide sufficient details for dose extraction or effect size computation. Trials were also excluded if comorbid conditions, mobility limitations, or insufficient participant characterization precluded meaningful participation in structured exercise interventions.

### 2.3. Information Sources and Search Strategy

A comprehensive search was conducted in PubMed (MEDLINE), Embase, and Web of Science Core Collection to identify eligible RCTs. Search terms combined concepts related to exercise (“physical exercise,” “physical activity,” “aerobic training,” “resistance training,” “multicomponent”), the target population (“dementia,” “Alzheimer*,” “cognitive impairment”), outcomes (“cognitive function,” “executive function,” “memory”), and study design (“randomized controlled trial,” “RCT”), using Boolean operators and MeSH terms where applicable. Filters were applied for human studies, the English language, and older adult populations when available, which may introduce potential language bias. The search was last updated on 20 December 2025. Reference lists of included trials and relevant reviews were additionally screened to identify further eligible studies [30]. Full electronic search strategies for all databases are provided in Supplementary Table S2.

### 2.4. Study Selection

**Figure 1:**
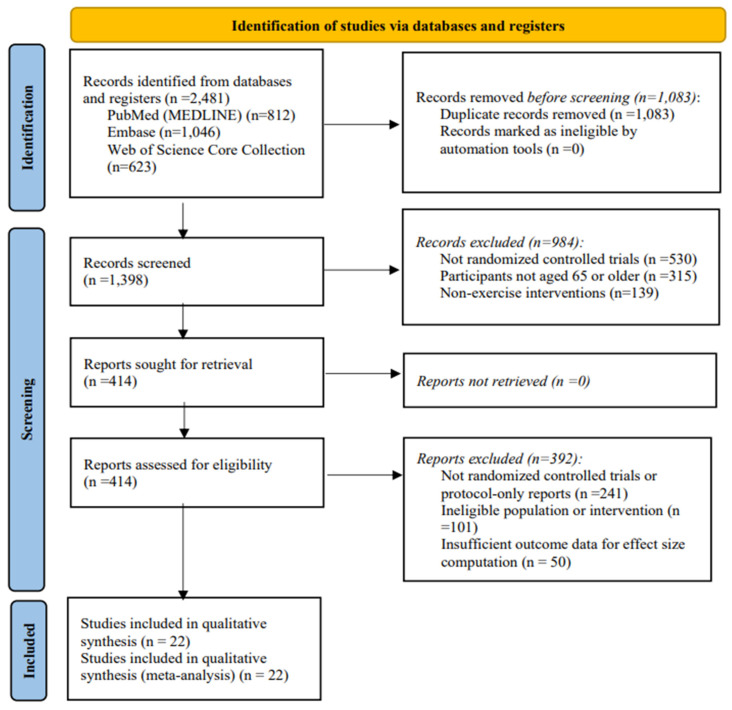
PRISMA Flow Diagram for Study Selection.

Titles and abstracts were screened against the eligibility criteria, followed by full-text assessment. Reasons for full-text exclusion were documented. The study selection process was conducted in accordance with PRISMA 2020 recommendations and is summarized in a PRISMA flow diagram (Figure 1) [31].

### 2.5. Data Extraction and Exercise Dose Quantification

Data were extracted using a standardized template, including author and year, country, intervention characteristics, comparator details, outcome instruments, outcome domain classification, and pre/post summary statistics.

To operationalize exercise dose, the following parameters were extracted or derived at the intervention-arm level: frequency (sessions/week), session duration (minutes/session), total number of sessions, weekly volume (minutes/week = frequency × minutes/session), total accumulated dose (minutes = total sessions × minutes/session), and program duration (weeks). When necessary, program duration was derived as total sessions divided by weekly frequency.

Interventions were further categorized as Physical only versus Cognitive plus Physical (e.g., dual-task training or exergaming with cognitive components). Dose categories based on weekly volume were prespecified as <90, 90–150, and >150 min per week, with an additional dichotomous comparison of ≥150 versus <150 min per week to reflect commonly used physical activity recommendations. These weekly volume categories were applied as operational thresholds to support comparability across trials rather than as dementia-specific prescription targets.

### 2.6. Outcome Domain Classification and Effect Size Computation

Outcomes were classified into three domains: cognitive function, functional ability, and mood and behavior. To provide an overall estimate of intervention effectiveness while avoiding unit-of-analysis errors, a single primary outcome per study was selected for the main pooled analysis to avoid unit-of-analysis errors. Although methodologically necessary, this approach may limit the inclusion of additional relevant outcome data. Cognitive outcomes were prioritized when available; if not reported, functional or mood and behavioral outcomes were selected. When trials reported multiple measures within the selected domain, global cognition (when available) and post-intervention time points were prioritized to yield one independent effect size per trial. Domain-specific subgroup analyses were subsequently conducted to explore potential differential effects across outcome domains. Detailed information on outcome selection, measurement instruments, sample sizes, and extracted effect sizes for each included trial is provided in Supplementary Table S3.

The effect size metric was Hedges’g (standardized mean difference with small-sample correction). Effect sizes were computed from unmatched groups using pre/post means and SDs, converting to standardized pre–post change differences. A pre–post correlation of r = 0.50 was assumed for effect size computation; sensitivity analyses examined r = 0.30 and r = 0.70. Reported change scores or adjusted post-intervention estimates were used when available; otherwise, effect sizes were derived from pre–post summary data using the assumed correlation [30,31].

Effect direction was harmonized such that positive g indicates improvement favoring the intervention. For scales where lower scores represent better status (e.g., depressive symptom scales, behavioral disturbance scales, mobility tests such as TUG), the sign was reversed so that improvement was consistently positive across outcomes.

### 2.7. Risk of Bias Assessment

Risk of bias was assessed using the Cochrane Risk of Bias 2 (RoB 2) framework, which evaluates five domains: bias arising from the randomization process, deviations from intended interventions, missing outcome data, measurement of the outcome, and selection of the reported result [32]. Each domain was judged as low risk, some concerns, or high risk of bias, and an overall risk-of-bias judgment was assigned in accordance with RoB 2 guidance [28]. Detailed domain-level and overall assessments for each included randomized controlled trial are presented in Supplementary Table S4. Given shared design characteristics of exercise-based randomized trials in dementia populations, broadly comparable patterns in domain-level risk-of-bias judgments were observed across studies, although variation in reporting and implementation was present. The classic fail-safe N was also calculated to estimate the number of additional null studies required to attenuate statistical significance.

### 2.8. Statistical Analysis

Random-effects models were estimated using the method of moments (MM) in Comprehensive Meta-Analysis (CMA). Between-study heterogeneity was quantified using τ^2^, Q, and I^2^ statistics. All analyses were independently replicated in Python (version 3.11), and restricted maximum likelihood (REML) estimates were examined as a replication and sensitivity check [31].

Primary meta-analysis. A random-effects meta-analysis was performed using one independent effect size per trial.

Subgroup analyses. Prespecified subgroup analyses examined differences by outcome domain, intervention type (Physical only versus Cognitive plus Physical), and dose categories based on weekly volume (<90, 90–150, >150 min per week; and ≥150 versus <150).

Meta-regression. Random-effects meta-regression analyses were conducted to examine whether commonly reported time-based dose indicators explained between-study heterogeneity. Candidate moderators included intervention duration (weeks), session length (minutes), weekly frequency, and total accumulated exercise dose. Given the conceptual and mathematical overlap among these variables, each dose indicator was entered into the meta-regression models separately to minimize collinearity. Multivariable models including multiple dose parameters were not prioritized, as preliminary correlation screening indicated substantial interdependence among time-based metrics.

Publication bias. Small-study effects and potential publication bias were assessed using funnel plots and Egger’s regression test; trim-and-fill analyses were conducted as supplementary analyses where appropriate [29].

Sensitivity analyses. Robustness of findings was examined using leave-one-out analyses, alternative assumed pre–post correlations (r = 0.30, 0.50, 0.70), and exclusion of studies judged to be at high risk of bias. Sensitivity analyses under alternative pre–post correlation assumptions and publication bias assessments are reported in Supplementary Table S5.

## 3. Results

### 3.1. Results of Study Selection

A total of 2481 records were identified through database searching, including 812 records from PubMed (MEDLINE), 1046 records from Embase, and 623 records from Web of Science Core Collection. After removing 1083 duplicate records, 1398 unique records remained for title and abstract screening. During screening, 984 records were excluded for not meeting the predefined eligibility criteria, primarily due to a non-randomized design, an ineligible population, or non-exercise interventions. Subsequently, 414 full-text reports were sought for retrieval and assessed for eligibility, and all reports were successfully retrieved. Of the 414 full-text reports assessed, 392 were excluded for the following reasons: non-randomized controlled trial design or protocol-only reports (n = 241), ineligible population or intervention (n = 101), and insufficient outcome data for effect size computation (n = 50). Ultimately, 22 randomized controlled trials met all inclusion criteria and were included in both the qualitative synthesis and the quantitative meta-analysis. The study selection process is summarized in Figure 1. A summary of full-text articles excluded after eligibility assessment is provided in Supplementary Table S6.

### 3.2. Study Characteristics

Across the 22 included randomized controlled trials, the total sample size was N = 1889, with a median of 86 participants per trial (range: 18–186). Exercise interventions were most commonly delivered at a median frequency of 2 sessions per week (IQR: 2–3), with a median session duration of 50 min per session (IQR: 45–60), over a median intervention period of 16 weeks (IQR: 12–26). The median prescribed weekly exercise volume was 120 min per week (IQR: 75–150), corresponding to a median total accumulated exercise dose of 720 min (IQR: 552–1560). Most interventions consisted of Physical-only exercise programs (n = 15), whereas a smaller subset incorporated explicit cognitive components alongside physical training (Cognitive + Physical; n = 7). Key characteristics of the included studies and exercise dose parameters are summarized in Table 1 [33,34,35,36,37,38,39,40,41,42,43,44,45,46,47,48,49,50,51,52,53,54].

### 3.3. Risk of Bias

**Figure 2:**
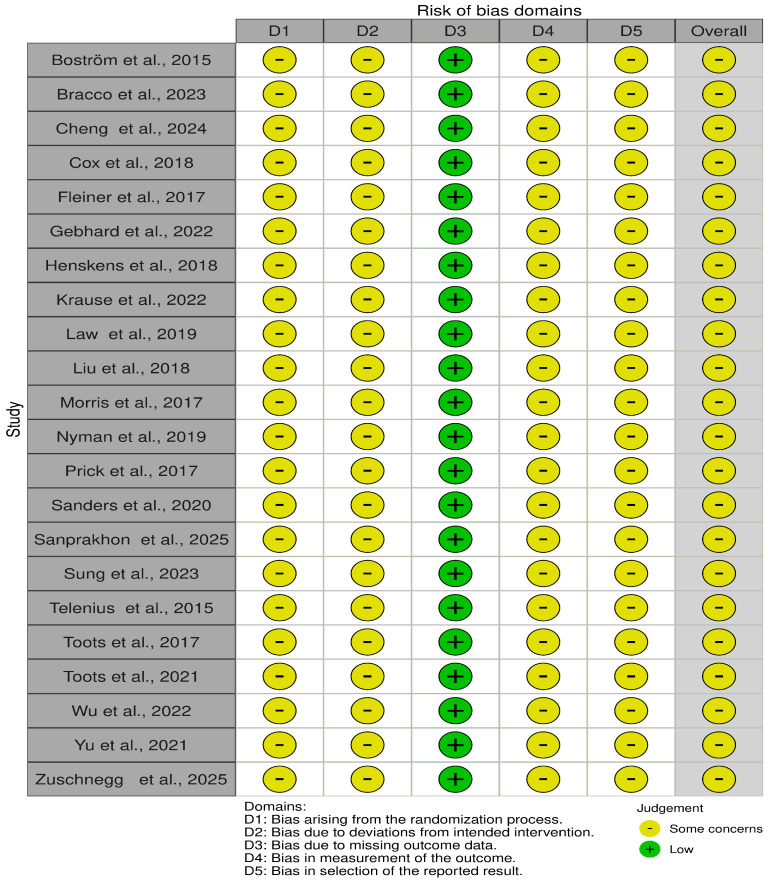
Domain-level risk of bias assessments for included randomized controlled trials using the Cochrane Risk of Bias 2 (RoB 2) tool. All studies included in this risk of bias assessment are cited in the reference list [33,34,35,36,37,38,39,40,41,42,43,44,45,46,47,48,49,50,51,52,53,54].

Risk of bias judgments for the included trials, assessed using the Cochrane Risk of Bias 2 (RoB 2) tool, are presented in Figure 2. Overall, the majority of trials were judged to have low risk of bias or some concerns across domains. Some concerns were most commonly related to incomplete reporting of randomization procedures, deviations from intended interventions, and aspects of outcome measurement, reflecting challenges frequently encountered in non-pharmacological exercise trials.

### 3.4. Overall Effect (Random- and Fixed-Effects)

**Figure 3:**
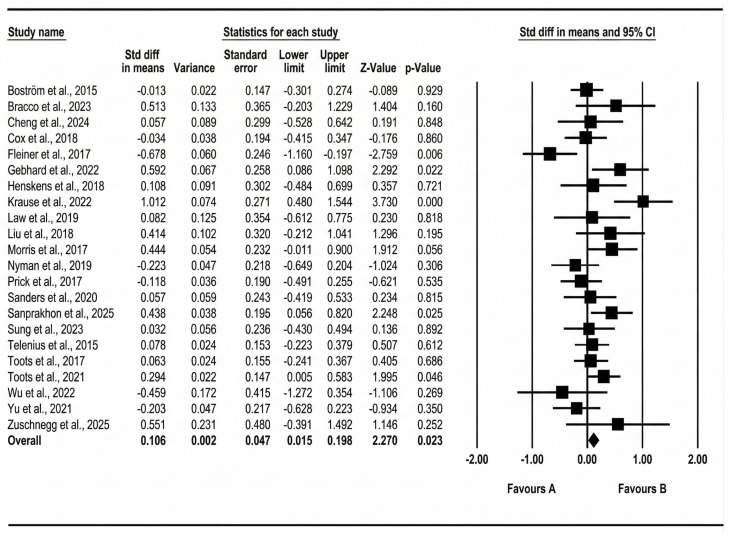
Forest plot of the overall pooled effect size across included studies [33,34,35,36,37,38,39,40,41,42,43,44,45,46,47,48,49,50,51,52,53,54].

**Figure 4:**
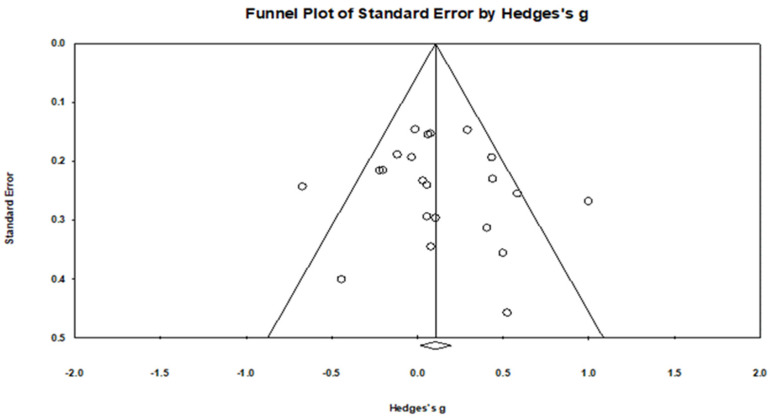
Funnel plot of standard error versus Hedges’ g for included studies.

A total of 22 effect sizes were synthesized. The primary outcomes selected for each trial and the corresponding extracted effect sizes used in the main meta-analysis are summarized in Supplementary Table S3. These outcomes represent the prespecified primary or most clinically relevant post-intervention measures from each study and form the basis of all pooled, subgroup, and sensitivity analyses reported below. Under a fixed-effect model, structured exercise interventions were associated with a small but statistically significant improvement (cognitive, functional, and mood or behavior outcomes) (Hedges’ g = 0.106, SE = 0.046, 95% CI 0.015–0.197, Z = 2.276, *p* = 0.023). However, given the presence of between-study heterogeneity, the random-effects model was considered the primary analytical framework. Under the random-effects model (method of moments), the pooled effect size remained similar in magnitude but with greater uncertainty and did not reach statistical significance (Hedges’ g = 0.117, SE = 0.070, 95% CI −0.021–0.254, Z = 1.660, *p* = 0.097). The corresponding forest plot and funnel plot are presented in Figure 3 and Figure 4, respectively.

#### Heterogeneity

There was evidence of statistically significant between-study heterogeneity (Q = 43.530, df = 21, *p* = 0.003), with moderate inconsistency (I^2^ = 51.757%). This level of heterogeneity suggests that intervention effects may vary across clinical and programmatic contexts, which may limit the direct generalizability of pooled estimates. The estimated between-study variance was τ^2^ = 0.052 (τ = 0.228), supporting the use of random-effects models and further exploration of potential sources of heterogeneity through subgroup and dose-based analyses.

### 3.5. Subgroup Analysis

Subgroup assignments and the selected study-level outcomes contributing to each pooled estimate are documented in Supplementary Table S3. Prespecified subgroup analyses were conducted to explore potential sources of heterogeneity related to intervention characteristics, outcome domains, and exercise dose.

#### 3.5.1. Training Type Subgroup (Cognitive + Physical vs. Physical-Only)

**Figure 5:**
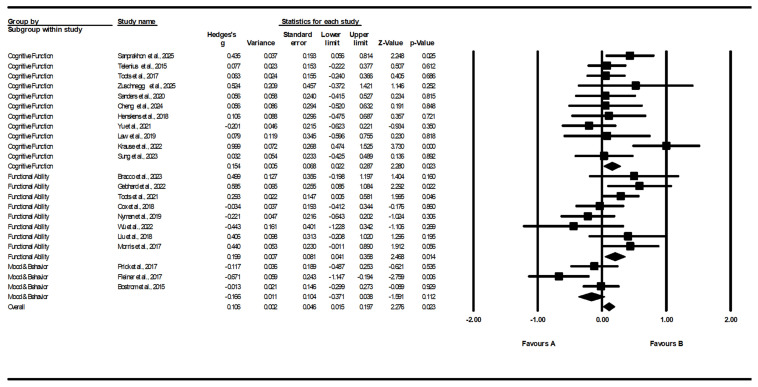
Subgroup pooled effects by intervention type [33,34,35,36,37,38,39,40,41,42,43,44,45,46,47,48,49,50,51,52,53,54].

Stratification by training type revealed substantial within-group variability and a small pooled effect under the fixed-effect model (overall fixed g = 0.106, *p* = 0.023). Individual studies demonstrated considerable dispersion in effect sizes, including one notably large positive estimate (Sanprakhon et al., 2025 [47]; g ≈ 2.035). Differences in outcome measurement instruments, such as the use of MoCA in this study, may also have contributed to variability in observed effect sizes. Given the limited number of trials within subgroups and the influence of extreme values, these findings should be interpreted as exploratory rather than indicative of a definitive difference between training types (Figure 5). One study exhibited an unusually large effect size; however, sensitivity analyses excluding this trial did not materially alter the overall pattern of results.

#### 3.5.2. Outcome-Domain Subgroup (Cognitive/Functional/Mood & Total Wellbeing)

In fixed-effect subgroup analyses by outcome domain, statistically significant pooled effects were observed for cognitive outcomes (k = 11; g = 0.154, SE = 0.068, 95% CI 0.022–0.287, *p* = 0.023) and functional outcomes (k = 8; g = 0.199, SE = 0.081, 95% CI 0.041–0.358, *p* = 0.014), whereas the mood or total wellbeing domain was not statistically significant (k = 3; g = −0.166, SE = 0.104, 95% CI −0.371–0.038, *p* = 0.122). The fixed-effect between-group comparison suggested significant subgroup differences (Q_between = 8.638, df = 2, *p* = 0.013).

**Figure 6:**
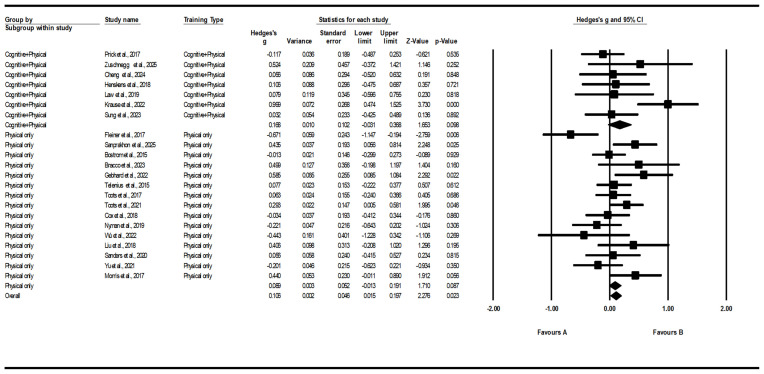
Subgroup pooled effects by outcome domain [33,34,35,36,37,38,39,40,41,42,43,44,45,46,47,48,49,50,51,52,53,54].

However, when using mixed-effects models to account for between-study heterogeneity, none of the domain-specific pooled effects reached conventional statistical significance (Cognitive: g = 0.171, *p* = 0.063; Functional: g = 0.199, *p* = 0.085; Mood or total wellbeing: g = −0.226, *p* = 0.211), and the between-group difference was no longer statistically significant (Q_between = 4.427, df = 2, *p* = 0.109) (Figure 6). Accordingly, domain-specific findings should be interpreted cautiously and viewed as hypothesis-generating rather than confirmatory. These findings were sensitive to the statistical model applied, and the limited number of studies within each domain reduces the robustness of between-domain comparisons.

#### 3.5.3. Total Exercise Dose Subgroup (Total Dose)

Subgroup analyses were conducted according to total accumulated exercise dose. Under the fixed-effect model, the overall pooled effect was small but statistically significant (Hedges’ g = 0.106, 95% CI 0.015–0.197, *p* = 0.023). The test for subgroup differences suggested heterogeneity across total dose categories (Q_between = 31.133, df = 15, *p* = 0.008), suggesting variation in observed effects across dose levels. The subgroup corresponding to a total dose of 1440 minutes (k = 6) demonstrated a small positive pooled effect (g = 0.117, 95% CI −0.021–0.254, *p* = 0.097).

**Figure 7:**
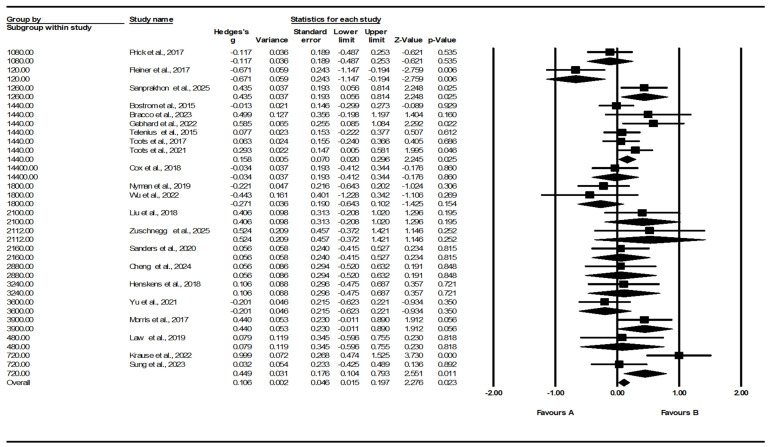
Subgroup pooled effects by total accumulated exercise dose [33,34,35,36,37,38,39,40,41,42,43,44,45,46,47,48,49,50,51,52,53,54].

Under the random-effects (mixed-effects) model, the overall pooled effect was not statistically significant (g = 0.117, 95% CI −0.033–0.165, *p* = 0.097). Although the between-group difference remained statistically significant (Q_between = 27.496, df = 15, *p* = 0.025), interpretation should be cautious because most total dose categories were represented by a single study (k = 1), resulting in imprecise subgroup estimates and limited inferential value (Figure 7). These findings should therefore be interpreted as exploratory rather than indicative of differential dose effects.

### 3.6. Publication Bias

Visual inspection of the funnel plot (Figure 4) did not suggest marked asymmetry. Egger’s regression intercept was 0.613 (SE = 1.057), providing no statistical evidence of small-study effects (t = 0.580, df = 20, two-tailed *p* = 0.568). The classic fail-safe N indicated that 12 additional null studies would be required to raise the observed *p*-value above α = 0.05, suggesting limited robustness to potential unpublished null findings. However, given the modest number of included studies, these assessments should be interpreted cautiously, as the absence of statistical evidence of bias does not necessarily indicate the absence of publication bias. Publication bias and small-study effect outputs based on the primary effect sizes are provided in Table S5c.

### 3.7. Meta-Regression

Meta-regression models were fitted using the trial-level effect sizes summarized in Supplementary Table S3, with dose moderators derived from the extracted intervention characteristics described in Table 1 and Section 2.5. To examine whether prespecified intervention dose characteristics explained variability in effect sizes, random-effects meta-regression analyses were conducted using three a priori moderators: total intervention duration (Weeks_total), session duration (Minutes_per_session), and total accumulated exercise dose (Total Dose).

In the primary random-effects (method of moments) meta-regression, the omnibus test of moderators was not statistically significant (Qm = 1.06, df = 3, *p* = 0.787), and none of the individual dose-related moderators were significantly associated with effect size: Weeks_total (β = 0.0043, 95% CI −0.0473 to 0.0428, *p* = 0.8552), Minutes_per_session (β = 0.0033, 95% CI −0.0045 to 0.0109, *p* = 0.3422), and Total Dose (β ≈ 0.0000, 95% CI −0.0003 to 0.0003, *p* = 0.8104). Substantial residual heterogeneity remained after adjustment for these moderators (Q = 40.94, df = 18, *p* = 0.0016), with τ^2^ = 0.0635 and I^2^ = 55.71%, indicating that a large proportion of between-study variability was not explained by these linear dose parameters. The proportion of explained heterogeneity was negligible (R^2^ analog = 0.00; computed value = −0.22), suggesting that inclusion of these moderators did not meaningfully reduce heterogeneity relative to the intercept-only model (Table 2).

**Figure 8:**
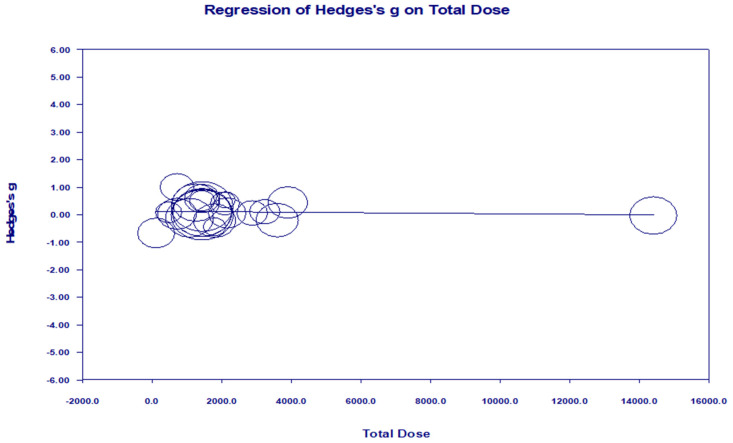
Meta-regression of Hedges’s g on total accumulated exercise dose across included studies.

A fixed-effect meta-regression conducted as a sensitivity analysis yielded consistent findings. The overall model test was not significant (Qm = 2.583, df = 3, *p* = 0.459), and none of the dose-related moderators were significantly associated with effect size (Weeks_total: *p* = 0.7515; Minutes_per_session: *p* = 0.136; Total Dose: *p* = 0.8400), supporting the robustness of the primary random-effects results (Figure 8). The absence of statistically significant associations should be interpreted cautiously, as it may reflect limited statistical power or restricted variability in prescribed exercise doses.

### 3.8. Sensitivity Analyses

Sensitivity analyses were conducted on the same set of trial-level effect sizes summarized in Supplementary Table S3; leave-one-out results are reported in Supplementary Table S5. Given the presence of an extreme positive effect estimate (Sanprakhon et al., 2025 [47]; g ≈ 2.035), sensitivity analyses were performed to assess the robustness of the pooled results to influential observations. Specifically, effect sizes exceeding ±2 standard deviations from the pooled distribution were identified, and leave-one-out analyses were conducted by re-estimating models after excluding the influential study. Because extreme values can disproportionately influence heterogeneity estimates and slope coefficients in meta-regression, these sensitivity analyses were used to contextualize the interpretation of dose–response findings and to evaluate the stability of the overall conclusions. The results of sensitivity analyses and publication bias assessments are summarized in Supplementary Table S5.

## 4. Discussion

### 4.1. Summary of Findings and the “Dose Paradox”

A key contribution of this review is that it not only highlights a modest overall effect of exercise on dementia outcomes but additionally shows the lack of consistent evidence for a linear dose–response relationship in this group. These findings challenge prevailing assumptions that higher exercise volume necessarily translates into proportionally greater benefits for individuals with dementia. The fixed-effect model of this 22-study meta-analysis of exercise-based intervention demonstrated a small but statistically significant pooled effect, while the random-effects model provided similar point estimates but with more uncertainty and a confidence interval that included the null value. The reason is that moderate between-study heterogeneity in the literature makes applying a random-effects model more suitable and cautious for the purposes of interpretation. Together, these findings imply that exercise may provide modest, context-specific benefits in dementia-related outcomes, in line with generalizable evidence that non-pharmacological and lifestyle interventions may slow functional decline, although with considerable variation across individuals and programs [3,11,12].

One of the key interpretive concerns in dose-focused syntheses is the “dose paradox”, where the overall pooled effects are modestly or statistically uncertain, but specific outcome domains or intervention types present less ambiguous signs of benefit. The current results agreed with this pattern. Cognitive and functional measures exhibited more consistently positive pooled effects when outcomes were stratified by domain than did mood or wellbeing outcomes. Although between-domain differences attained statistical significance under fixed-effect assumptions, these differences attenuated and ceased to be statistically significant once between-study heterogeneity was controlled for using mixed-effects models. This pattern indicates that both outcome selection and measurement domain influence the intervention signal seen. Domain-specific responsiveness of this nature has been observed often in dementia trials with different sensitivity, measurement properties, and clinical relevance of cognitive vs. functional vs. psychosocial endpoints [4,11,19].

### 4.2. Why Linear Meta-Regression May Fail: Non-Linearity and Threshold Behavior

Standard meta-regression approaches typically impose a linear dose–response structure. However, the inability of linear dose indicators to explain between-study heterogeneity in the present analyses should not be interpreted as evidence that exercise dose is unimportant in dementia. Rather, these findings are consistent with the possibility that exercise effects in this population may follow non-linear dynamics, such as threshold or saturating patterns, although this possibility was not directly tested in the present study. From a biological perspective, relatively modest increases from very low baseline activity levels may yield disproportionate benefits through improvements in cerebral perfusion, metabolic regulation, and neuromuscular function. In contrast, additional increments beyond a certain exposure level may confer diminishing returns, particularly in older adults with neurodegenerative disease [14,15,17].

Clinical and contextual factors further support a non-linear interpretation. Dementia severity, comorbid frailty, polypharmacy, and limited physiological reserve can constrain achievable training adaptations, producing a threshold-then-plateau pattern rather than a monotonic dose–response relationship. Such dynamics may be more readily detected using clinically meaningful dose categories or minimum effective exposure concepts than by estimating a single continuous slope across heterogeneous trials [11,19,22]. Accordingly, the assumption that “more is always better” may not universally apply in exercise interventions. Instead, the available evidence is more compatible with the view that achieving a feasible minimum effective exposure may be more important than maximizing prescribed dose, particularly when study-level doses cluster within a restricted range and intervention content varies substantially across trials.

### 4.3. Ceiling Effects and Adherence: Why Higher Planned Dose May Not Translate to Higher Received Dose

A second explanation for the weak linear associations observed is the presence of ceiling effects at both the participant and program levels. Individuals with dementia may exhibit reduced trainability due to neurodegeneration, sarcopenia, and cardiovascular limitations, leading to early saturation of measurable improvements even when exercise exposure is increased [11,16]. In parallel, programs prescribing higher exercise doses often experience lower adherence and higher dropout rates, particularly when supervision is limited or when cognitive impairment compromises self-management capacity. When trial reports emphasize prescribed dose rather than dose actually received, such discrepancies introduce dose misclassification, which can bias meta-regression estimates toward the null [11,21]. Reporting of adherence and actual received dose was inconsistent across trials, limiting the ability to systematically examine their influence on pooled estimates. Variability in participant engagement and participation intensity may further influence the relationship between prescribed and received exercise dose, although these factors were not formally examined as moderators in the present analysis. Such variability may contribute to non-linear response patterns, including hormetic relationships, in which both insufficient and excessive exercise exposure may limit observed benefits.

Future trials and evidence syntheses should therefore prioritize standardized reporting of adherence-related indicators, including attendance, compliance, and fidelity to prescribed intensity. Consideration of “effective dose” metrics that incorporate attendance-weighted exposure, rather than nominal prescription alone, may provide a more accurate representation of intervention exposure and its relationship with outcomes.

### 4.4. Heterogeneity Beyond Dose: Intervention Composition and Outcome Domain

Exercise dose represents only one dimension of intervention variability. The observed domain-dependent effects suggest that intervention composition and outcome selection may be as influential as total exposure time. Differences in training content, such as multicomponent functional programs, aerobic emphasis, or dual-task cognitive–motor elements, as well as the choice of outcome measures, including cognitive tests, functional scales, or mood indices, may substantially shape the observed intervention signal [4,11,12].

Additional sources of heterogeneity likely include participant-level factors, such as dementia subtype, baseline severity, comorbidities, and caregiver support, as well as delivery characteristics, including supervision level, group-based versus home-based formats, and concurrent care components or medication changes [11,21]. Taken together, these considerations reinforce that exercise “dose” should be interpreted as one component within a multidimensional intervention package rather than as an isolated determinant of effect. Variation in cognitive outcome measures may also influence the magnitude of observed effects. Instruments such as MMSE may exhibit limited sensitivity to short-term changes, particularly in older populations, whereas alternative measures (e.g., MoCA) may capture different domains of cognitive function.

### 4.5. Interpreting Model 1: Explanatory Power and Collinearity Constraints

In the primary random-effects meta-regression model (CMA Model 1, method of moments), Weeks_total, Minutes_per_session, and Total Dose did not explain between-study variability (Q_m (3) = 1.06, *p* = 0.8178), and none of the individual coefficients reached statistical significance (all *p* ≥ 0.411). The proportion of explained between-study variance was effectively zero (R^2^ analog = 0.00; computed value = −0.22), while meaningful residual heterogeneity persisted (I^2^ = 55.71%; Q (18) = 42.90, *p* = 0.0013). These findings indicate that, within the current evidence base, linear combinations of these dose indicators do not account for observed heterogeneity in effect sizes.

From a methodological perspective, interpretation should also consider collinearity among dose variables. Total Dose is a derived construct based on time-related components, and simultaneous inclusion of derived and component metrics can increase collinearity and destabilize regression estimates, particularly when the number of available studies is limited [22,31]. Even under these constraints, the overall conclusion remains clear: the present data do not support a detectable linear dose–response gradient across trials.

### 4.6. Influence, Leverage, and Robustness

Although no single study appeared to dictate the overall conclusions, the dataset included trials with comparatively large effect estimates and or larger standard errors, which can influence pooled results and regression fits. Influence diagnostics, such as leave-one-out analyses, Baujat plots, or model-based influence outputs, are therefore important for transparent inference, particularly when heterogeneity is moderate and moderator effects are non-significant [31]. Reporting pooled estimates with and without high-leverage studies can strengthen robustness claims and reduce the risk of overinterpreting apparent subgroup patterns.

### 4.7. Clinical Implications

At a clinical level, these results substantiate exercise as a potentially beneficial adjunct strategy in people with dementia, especially when considering that mean effects are modest and differ by outcome domain and program settings. In progressive neurodegenerative diseases, even small-scale memory or functional preservation could yield considerable clinical value through lessened loss of independence, caretaker burden, and an improved quality of life.

From a practical standpoint, based on our findings, perhaps it is better to focus on practical and sustainable exercise programs that are feasible rather than to emphasize unlimited dose escalation. Instead of simply optimizing the volume of prescribed exercise, clinicians and program designers may need to pay more attention to adherence, supervision, and the fit between the intervention content, patient’s level of ability, and planned outputs [3,11,21], particularly functional activities pertinent to activities of daily living. At the level of health care delivery, it is emphasized that the effectiveness of exercise interventions in dementia care is determined less by the potential dose prescribed and more by the degree to which appropriate, supervised, and context-sensitive programs are available in the real-world clinical and community settings, although these factors were not directly examined in the present analyses.

### 4.8. Dose Indicators and the Absence of a Linear Dose–Response Signal

Although exercise has frequently been reported to benefit cognition in older adults with cognitive impairment and dementia [4,11,19], the present meta-regression analyses did not support a linear dose–response association when total intervention duration (Weeks_total), session duration (Minutes_per_session), and total accumulated exercise dose (Total Dose) were modeled simultaneously. In the primary random-effects meta-regression, the omnibus moderator test was not statistically significant (Q_m (3) = 1.06, *p* = 0.8178), and all regression coefficients were close to zero. Substantial residual heterogeneity persisted (I^2^ = 55.71%), indicating that between-trial differences are likely driven by factors beyond these time- and volume-based dose indicators.

Importantly, the absence of a detectable linear association should not be interpreted as evidence that exercise dose is irrelevant. Several alternative explanations remain plausible. First, commonly reported dose metrics may lack sensitivity to capture biologically and behaviorally relevant aspects of training exposure, such as intensity fidelity, progression, or adherence-weighted dose [11,21]. Second, dose–response relationships in dementia may be non-linear, with threshold or ceiling effects whereby benefits accrue within a feasible exposure range but do not increase proportionally at higher prescribed doses [14,15,22]. Third, intervention content may modify the relationship between dose and outcomes, particularly in multicomponent or combined cognitive–exercise programs, which may confer benefits under specific contextual or implementation conditions [24,27].

Consistent with potential non-linearity or effect modification, subgroup analyses by total accumulated exercise dose revealed statistically significant between-group differences under both fixed- and mixed-effects assumptions. However, most dose categories were represented by single studies, limiting precision and generalizability. Accordingly, these findings should be viewed as hypothesis-generating. Future research would benefit from pre-specifying clinically meaningful dose categories to avoid sparse strata and from applying non-linear modeling approaches, such as spline-based meta-regression, while avoiding simultaneous inclusion of mathematically redundant dose indicators [22,31]. The absence of a linear dose–response relationship suggests that commonly reported time-based dose indicators may be insufficient to capture key determinants of exercise responsiveness in dementia. Rather than total exercise volume alone, qualitative and contextual factors, including supervision, task specificity, adherence, and participant engagement, may exert a more influential role in shaping intervention outcomes [11,21]. From a practical perspective, these findings support the feasibility of implementing moderate-frequency, supervised exercise programs without requiring high accumulated volumes, and underscore the importance of future trials adopting adaptive or dose-ranging designs that better reflect real-world clinical and community settings [4,19,31].

### 4.9. Limitations

Several limitations should be acknowledged. Moderate between-study heterogeneity persisted across analyses, and potential misclassification of exercise dose may have occurred due to reliance on prescribed rather than received exposure. Data within many dose strata were sparse, limiting the precision of subgroup estimates, particularly for subgroup analyses based on total accumulated dose, where some categories included only a single study and therefore provide limited inferential value and should be interpreted as exploratory. In addition, statistical power for meta-regression was constrained by the number of available trials, increasing the likelihood of type II error and limiting the ability to detect true dose–response associations. These limitations are common in behavioral and lifestyle intervention meta-analyses and underscore the need for improved harmonization of dose metrics, adherence reporting, and intervention characterization in future exercise trials involving individuals with dementia [11,22,31]. Furthermore, exercise dose was operationalized primarily using time-based indicators rather than multidimensional exposure measures (e.g., session duration, weekly volume, accumulated exposure), without consistent reporting of intensity metrics such as relative load or metabolic equivalents. The absence of standardized reporting of received dose further limits the precision of exposure classification and may attenuate detectable dose–response associations.

Although a formal GRADE assessment was not conducted, the overall certainty of the evidence should be considered limited, which reduces the strength of clinical implications derived from the present findings. This judgment reflects residual between-study heterogeneity, imprecision of pooled estimates under random-effects models, and substantial variability in intervention characteristics and outcome measures across trials. Furthermore, many studies included in the review were of limited sample size, with domain-specific questions of risk of bias, limiting confidence in the magnitude and consistency of observed effects. As such, the results of the present review must be regarded with appropriate caution. These methodological limitations are in line with the risk-of-bias profile observed across included trials, in which most studies were judged to have some concerns, mostly on account of reporting-related limitations and due to the difficulty in blinding in exercise-based interventions.

## 5. Conclusions

This meta-analytic work synthesizes evidence from several randomized studies that describe the magnitude, variability, and dose-modifying effects of exercise interventions in patients with dementia. Interestingly, linear dose attributes of program duration, session length, and total accumulated exercise dose did not appear to differ in effect sizes across the trials based on available randomized data. Altogether, these findings indicate that response to exercise in dementia is unlikely to incorporate a more specific linear dose–response effect and is more based on non-linear parameters and intervention types beyond timing-dependent dose measurements. But future work should concentrate on standardized and transparent reporting of prescribed and received exercise exposure, adherence-sensitive, adaptive design recommendations, and development of achievable minimum dosages that will maximize cognition and function among that population. Importantly, lack of an apparent direct dose–response relationship should not be accepted as evidence against exercise for dementia; rather, the effect of exercise may be influenced by feasibility, quality of implementation, and contextual factors not captured by time-dependent dose statistics.

## Figures and Tables

**Table 1:** Characteristics of included trials and exercise dose parameters (FITT framework).

Study	Country	Population/Diagnosis	n (Intervention/Control)	Intervention Type	Modality	Frequency (Sessions/wk)	Session (min)	Duration (Weeks)	Weekly Volume (min/wk)	Total Dose (min)
Boström et al., 2015 [33]	Sweden	Dementia (residential care)	93/93	Physical only	High-intensity functional exercise program	2	45	16	90	1440
Bracco et al., 2023 [34]	France	Dementia	15/16	Physical only	Tango physical exercise	2	60	12	120	1440
Cheng et al., 2024 [35]	Taiwan	SCD/cognitive impairment	33/17	Cognitive + Physical	Physical + cognitive training	2	60	24	120	2880
Cox et al., 2018 [36]	Australia	At risk of AD	51/55	Physical only	Physical activity program	3	50	96	150	14400
Fleiner et al., 2017 [37]	Germany	Dementia (hospital setting)	35/35	Physical only	Short-term exercise program	3	20	2	60	120
Gebhard et al., 2022 [38]	Austria	Dementia	34/29	Physical only	Otago Exercise Program	2	60	12	120	1440
Henskens et al., 2018 [39]	Netherlands	Mild-to-moderate cognitive impairment	22/22	Cognitive + Physical	Multicomponent exercise ± ADL training	3	45	24	135	3240
Krause et al., 2022 [40]	USA	Cognitive impairment/risk	26/37	Cognitive + Physical	Yoga + Memory Enhancement Training (MET)	1	60	12	60	720
Law et al., 2019 [41]	Hong Kong	MCI	16/16	Cognitive + Physical	Functional task exercise + exercise training	1	60	8	60	480
Liu et al., 2018 [42]	China	Dementia	20/20	Physical only	Passive finger movement exercise	7	25	12	175	2100
Morris et al., 2017 [43]	USA	Early AD	39/37	Physical only	Aerobic exercise vs. stretching/toning	5	30	26	150	3900
Nyman et al., 2019 [44]	United Kingdom	Dementia (community-dwelling)	42/43	Physical only	Tai Chi	1	90	20	90	1800
Prick et al., 2017 [45]	Netherlands	Dementia	57/54	Cognitive + Physical	Exercise + psychoeducation + communication skills + pleasant activities	3	30	12	90	1080
Sanders et al., 2020 [46]	Netherlands	Dementia	39/30	Physical only	Folk dance	3	30	24	90	2160
Sanprakhon et al., 2025 [47]	Thailand	Mild behavioral impairment/older adults	56/52	Physical only	Folk dance (with cognitive stimulation program)	2	90	7	180	1260
Sung et al., 2023 [48]	Taiwan	MCI/mild dementia	36/36	Cognitive + Physical	Multi-domain cognitive function training (MCFT)	3	30	8	90	720
Telenius et al., 2015 [49]	Norway	Dementia	87/83	Physical only	High Intensity Functional Exercises (HIFE)	2	60	12	120	1440
Toots et al., 2017 [50]	Sweden	Dementia (nursing home)	84/82	Physical only	High-intensity functional exercise program (HIFE)	2	45	16	90	1440
Toots et al., 2021 [51]	Sweden	Dementia (nursing home)	93/93	Physical only	HIFE	2	45	16	90	1440
Wu et al., 2023 [52]	South Korea	Dementia/cognitive impairment	13/11	Physical only	Exergame training vs. aerobic exercise	3	50	12	150	1800
Yu et al., 2021 [53]	USA	Mild-to-moderate AD dementia	64/32	Physical only	Moderate-intensity cycling vs. light stretching	3	50	24	150	3600
Zuschnegg et al., 2025 [54]	Austria	Mild-to-moderate AD	9/9	Cognitive + Physical	Tablet-based multimodal vs. paper-and-pencil	4	22	24	88	2112

**Table 2:** Random-effects meta-regression models for exercise dose parameters (n = 22).

Model	Predictors (β)	β (SE)	*p*	Qm (df)	p (Qm)	R^2^
Model 1 (Primary; CMA, random [MM])	Intercept	−0.0629 (0.2430)	0.7959	1.06 (3)	0.7867	0.00
	Weeks total	0.0043 (0.0237)	0.8552			
	Minutes per session	0.0033 (0.0040)	0.3422			
	Total Dose	0.0000 (0.0002)	0.8104			
Model 1 (Sensitivity; CMA, fixed)	Intercept	−0.0258 (0.1637)	0.8746	2.580 (3)	0.4594	—
	Weeks total	−0.0049 (0.0153)	0.7515			
	Minutes per session	0.0035 (0.0026)	0.1362			
	Total Dose	0.0000 (0.0001)	0.84			

Note. Random-effects Model 1 residual heterogeneity: I^2^ = 56.03%, Q (18) = 40.94, *p* = 0.0016, τ^2^ = 0.0632; Null model heterogeneity: I^2^ = 51.76%, Q (21) = 43.53, *p* = 0.0027, τ^2^ = 0.0518.

## Data Availability

No new data were created or analyzed in this study. Data sharing is not applicable to this article.

## Supplementary Materials

The following supporting information can be downloaded at: https://www.mdpi.com/article/10.3390/healthcare14050689/s1, Table S1. PRISMA 2020 Checklist; Table S2. Full electronic search strategies for all databases; Table S3. Outcome Selection and Effect Size Extraction; Table S4. Risk of Bias Assessment Using the Cochrane RoB 2 Tool; Table S5. Sensitivity and Publication Bias Analyses; Table S6. Excluded Full-Text Studies with Reasons.

## Abbreviations

AD	Alzheimer’s disease
BPSD	Behavioral and psychological symptoms of dementia
CI	Confidence interval
CMA	Comprehensive Meta-Analysis
IQR	Interquartile range
MM	Method of moments
PRISMA	Preferred Reporting Items for Systematic Reviews and Meta-Analyses
PROSPERO	International Prospective Register of Systematic Reviews
RCT	Randomized controlled trial
RoB 2	Risk of Bias 2 tool
SD	Standard deviation
SE	Standard error
SMD	Standardized mean difference
