# Supplementary material for: Exercise Interventions for Cognitive and Functional Outcomes in Dementia: A Systematic Review and Meta-Analysis Exploring Dose Metrics, Heterogeneity, and Implementation-Relevant Factors
Source: Healthcare (Basel). 2026 Mar 9;14(5):689. doi: 10.3390/healthcare14050689 (PMC12985021; doi:10.3390/healthcare14050689)
Supplement: Supplementary file 1 [file healthcare-14-00689-s001.zip › Table S1. PRISMA 2020 Checklist.pdf]

Table S1. PRISMA 2020 Checklist

Section and Topic	Item No.	Checklist Item	Location where the item is reported
**TITLE**	1	Identify the report as a systematic review, meta-analysis, or both.	Title/ p. 1
**ABSTRACT**	2	Provide a structured summary of the review, including background, objectives, data sources, eligibility criteria, synthesis methods, results, limitations, and conclusions.	Abstract/ p. 1
**INTRODUCTION**	3	Describe the rationale for the review in the context of existing knowledge.	Section 1. Introduction/ pp. 1-2
	4	Provide an explicit statement of the objectives or questions addressed by the review.	Section 1. Introduction/ p. 3
**METHODS**	5	Specify the inclusion and exclusion criteria and how studies were grouped for syntheses.	Section 2.2 Eligibility Criteria/ p. 3
	6	Specify all databases, registers, websites, and other sources searched or consulted, and the date of the last search.	Section 2.3 Information Sources and Search Strategy/ p. 3
	7	Present the full search strategies for all databases and sources, including any limits used.	Section 2.3 Information Sources and Search Strategy; Supplementary Table S2/ p. 3
	8	Specify the methods used to decide whether a study met the inclusion criteria, including the number of reviewers and whether they worked independently.	Section 2.4 Study Selection/ p. 4
	9	Specify the methods used to collect data from reports, including the number of	Section 2.5 Data Extraction and Exercise Dose

Section and Topic	Item No.	Checklist Item	Location where the item is reported
		reviewers and whether they worked independently.	Quantification/ p. 4
	10a	List and define all outcomes for which data were sought.	Section 2.6 Outcome Domain Classification and Effect Size Computation/ p. 4
	10b	Describe assumptions made about missing or unclear information.	Section 2.6 Outcome Domain Classification and Effect Size Computation/ p. 4
	11	Specify the methods used to assess risk of bias in the included studies.	Section 2.7 Risk of Bias Assessment/ p. 5
	12	Specify the effect measure(s) used for each outcome.	Section 2.6 Outcome Domain Classification and Effect Size Computation/ p. 4
	13a	Describe the processes used to decide which studies were eligible for each synthesis.	Section 2.6 Outcome Domain Classification and Effect Size Computation/ p. 4
	13b	Describe any methods required to prepare data for synthesis.	Section 2.6 Outcome Domain Classification and Effect Size Computation/ p. 4
	13c	Describe any methods used to tabulate or visually display results.	Section 2.6 Outcome Domain Classification and Effect Size Computation/ p. 4
	13d	Describe the methods used to synthesize results and the rationale for the chosen methods.	Section 2.8 Statistical Analysis/ p. 5
	13e	Describe methods used to explore possible causes of heterogeneity.	Section 2.8 Statistical Analysis/ p. 5

Section and Topic	Item No.	Checklist Item	Location where the item is reported
	13f	Describe any sensitivity analyses conducted to assess robustness of the results.	Section 2.8 Statistical Analysis/ p. 5
	14	Describe any methods used to assess risk of bias due to missing results (publication bias).	Section 2.7 Risk of Bias Assessment/ p. 5
	**15**	Describe any methods used to assess certainty (or confidence) in the body of evidence.	Not formally assessed; addressed narratively in Discussion (Section 4.9 Limitations)/ p. 19
**RESULTS**	16a	Describe the results of the search and selection process.	Section 3.1 Study Selection/ pp. 5-6
	16b	Cite studies that appeared to meet inclusion criteria but were excluded, and explain why.	Section 3.1 Study Selection; Supplementary Table S6/ p. 6
	17	Cite each included study and present its characteristics.	Section 3.2 Study Characteristics/ pp. 6-8
	18	Present assessments of risk of bias for each included study.	Section 3.3 Risk of Bias/ pp. 8-9
	19	Present summary statistics and effect estimates for each outcome.	Section 3.4 Overall Effects/ pp. 9-10
	20a	Summarize characteristics and risk of bias among studies contributing to each synthesis.	Sections 3.2 and 3.3/ pp. 6-8 and pp. 8-9
	20b	Present results of all statistical syntheses, including measures of heterogeneity.	Sections 3.4.1 and 3.5/ pp. 10-12
	20c	Present results of investigations of heterogeneity.	Sections 3.5 and 3.7/ 10-12 and 13-14
	20d	Present results of sensitivity analyses.	Section 3.8 Sensitivity Analyses/ pp. 14-15

Section and Topic	Item No.	Checklist Item	Location where the item is reported
	21	Present assessments of risk of bias due to missing results.	Section 3.6 Publication Bias/ p. 13
	**22**	Present assessments of certainty (or confidence) in the body of evidence for each outcome.	Not formally assessed; limitations related to certainty discussed narratively in Discussion (Section 4.9 Limitations) / p. 19
**DISCUSSION**	23a	Provide a general interpretation of the results in the context of other evidence.	Section 4. Discussion/ pp. 15-19
	23b	Discuss limitations of the evidence included in the review.	Section 4.9 Limitations/ p. 19
	23c	Discuss limitations of the review processes used.	Section 4.9 Limitations/ p. 19
	23d	Discuss implications of the results for practice, policy, and future research.	Sections 4.7 Clinical Implications and 4.8 Dose Indicators/ pp. 17-18 and pp. 18-19
**OTHER INFORMATION**	24a	Provide registration information for the review.	Section 2.1 Protocol and Reporting/ p. 3
	24b	Indicate where the review protocol can be accessed.	Section 2.1 Protocol and Reporting/ p. 3
	25	Describe sources of financial or non-financial support.	Funding/ p. 20
	26	Declare competing interests.	Conflicts of Interest/ p. 20
	27	Describe availability of data, analytic code, and materials.	Data Availability Statement; Supplementary Materials/ p. 20

Note: This checklist documents adherence of the present systematic review and meta-analysis to the PRISMA 2020 reporting guidelines, with corresponding section references

provided for transparency and completeness.
