# Supplementary material for: Exercise Interventions for Cognitive and Functional Outcomes in Dementia: A Systematic Review and Meta-Analysis Exploring Dose Metrics, Heterogeneity, and Implementation-Relevant Factors
Source: Healthcare (Basel). 2026 Mar 9;14(5):689. doi: 10.3390/healthcare14050689 (PMC12985021; doi:10.3390/healthcare14050689)
Supplement: Supplementary file 1 [file healthcare-14-00689-s001.zip › Table S2. Full electronic search strategies for all databases.pdf]

Table S2. Full electronic search strategies for all databases

**Database**	**Search strategy (full search string)**	**Filters / Limits**	**Last search date**
**PubMed (MEDLINE)**	(“Dementia”[Mesh] OR “Alzheimer Disease”[Mesh] OR dementia[Title/Abstract] OR alzheimer*[Title/Abstract]) AND (“Exercise”[Mesh] OR “Exercise Therapy”[Mesh] OR exercise[Title/Abstract] OR “physical activity”[Title/Abstract] OR training[Title/Abstract]) AND (“Cognition”[Mesh] OR “Cognition Disorders”[Mesh] OR cognitive[Title/Abstract] OR functional[Title/Abstract] OR behavior*[Title/Abstract])	Humans; Randomized Controlled Trial	20 December 2025
**Embase (Elsevier)**	(‘dementia’/exp OR ‘alzheimer disease’/exp OR dementia:ti,ab OR alzheimer*:ti,ab) AND (‘exercise’/exp OR ‘exercise therapy’/exp OR exercise:ti,ab OR ‘physical activity’:ti,ab OR training:ti,ab) AND (‘cognition’/exp OR ‘cognitive defect’/exp OR cognitive:ti,ab OR functional:ti,ab OR behavior*:ti,ab)	Human; Randomized controlled trial	20 December 2025
**Web of Science Core Collection**	TS = ((dementia OR alzheimer*) AND (exercise OR “physical activity” OR training) AND (cognitive OR functional OR behavioral OR behaviour*))	SCI-EXPANDED; SSCI	20 December 2025

Note: The complete search strategies were developed a priori and tailored to the indexing structure of each database. The final search was conducted on the dates specified, without language restrictions.
