# Supplementary material for: Exercise Interventions for Cognitive and Functional Outcomes in Dementia: A Systematic Review and Meta-Analysis Exploring Dose Metrics, Heterogeneity, and Implementation-Relevant Factors
Source: Healthcare (Basel). 2026 Mar 9;14(5):689. doi: 10.3390/healthcare14050689 (PMC12985021; doi:10.3390/healthcare14050689)
Supplement: Supplementary file 1 [file healthcare-14-00689-s001.zip › Table S3. Outcome Selection and Effect Size Extraction.pdf]

Table S3. Outcome Selection and Effect Size Extraction

Study	Selected domain	Selected instrument	Time point	n (I/C)	Hedges' g	Extraction note
Boström et al., 2015 [33]	Cognitive	MMSE	Post-intervention (end of week 16)	93 / 93	−0.013	Selected cognitive outcome as the only reported global cognitive measure; post-intervention endpoint used.
Bracco et al., 2023 [34]	Cognitive	MMSE	Post-intervention (end of week 12)	15 / 16	0.513	Multiple cognitive measures were reported; MMSE was selected as the global cognition indicator per prespecified hierarchy. Post-intervention endpoint used.
Cheng et al., 2024 [35]	Cognitive	Executive function / TMT-B	Post-intervention (end of week 24)	33 / 17	0.057	A task-specific executive function measure (TMT-B) was reported as the primary cognitive outcome; post-intervention values were selected.
Cox et al., 2018 [36]	Cognitive	MMSE	Post-intervention (end of week 96)	51 / 55	−0.034	MMSE was the sole cognitive outcome reported; post-intervention assessment selected for consistency.
Fleiner et al., 2017 [37]	Cognitive	MMSE	Post-intervention (end of week 2)	35 / 35	−0.678	Although behavioral outcomes were also reported, MMSE was selected as the primary cognitive outcome according to the prespecified hierarchy.
Gebhard et al., 2022 [38]	Cognitive	MMSE	Post-intervention (end of week 12)	34 / 29	0.592	Multiple cognitive measures were available; MMSE was selected as the global cognitive outcome. Post-intervention endpoint used.
Henskens et al., 2018 [39]	Cognitive	MMSE	Post-intervention (end of week 24)	22 / 22	0.108	MMSE was the only reported cognitive outcome and was selected at the immediate post-intervention time point.

Study	Selected domain	Selected instrument	Time point	n (I/C)	Hedges’ g	Extraction note
Krause et al., 2022 [40]	Cognitive	MMSE	Post-intervention (end of week 12)	26 / 37	1.012	Multiple cognitive outcomes were reported; MMSE was selected as the primary global cognition measure at post-intervention.
Law et al., 2019 [41]	Cognitive	Executive function / TMT-B	Post-intervention (end of week 8)	16 / 16	0.082	Executive function (TMT-B) was the primary cognitive outcome reported; post-intervention values were selected.
Liu et al., 2018 [42]	Cognitive	MMSE	Post-intervention (end of week 12)	20 / 20	0.414	MMSE was the sole cognitive outcome reported; post-intervention endpoint used.
Morris et al., 2017 [43]	Cognitive	MMSE	Post-intervention (end of week 26)	39 / 37	0.444	Multiple cognitive domains were assessed; MMSE was selected as the global cognition outcome per hierarchy.
Nyman et al., 2019 [44]	Functional	TUG	Post-intervention (end of week 20)	42 / 43	−0.223	No cognitive outcome was reported; a functional mobility outcome (TUG) was selected according to the prespecified hierarchy.
Prick et al., 2017 [45]	Cognitive	MMSE	Post-intervention (end of week 12)	57 / 54	−0.118	MMSE was the only reported cognitive outcome; post-intervention assessment used.
Sanders et al., 2020 [46]	Cognitive	ADAS-Cog	Post-intervention (end of week 24)	39 / 30	0.057	ADAS-Cog was reported as the primary cognitive outcome; post-intervention values selected.
Sanprakhon et al., 2025 [47]	Cognitive	MoCA	Post-intervention (end of week 7)	56 / 52	0.438	MoCA was the primary and only cognitive outcome reported; post-intervention assessment selected.

Study	Selected domain	Selected instrument	Time point	n (I/C)	Hedges' g	Extraction note
Sung et al., 2023 [48]	Cognitive	MMSE	Post-intervention (end of week 8)	36 / 36	0.032	MMSE was the sole cognitive outcome reported; post-intervention endpoint used.
Telenius et al., 2015 [49]	Cognitive	MMSE	Post-intervention (end of week 12)	87 / 83	0.078	MMSE was the primary cognitive outcome reported; post-intervention values selected.
Toots et al., 2017 [50]	Cognitive	ADAS-Cog	Post-intervention (end of week 16)	84 / 82	0.063	ADAS-Cog was reported as the primary cognitive outcome; post-intervention endpoint selected.
Toots et al., 2021 [51]	Cognitive	MMSE	Post-intervention (end of week 16)	93 / 93	0.295	MMSE was the sole reported cognitive outcome; post-intervention assessment selected.
Wu et al., 2023 [52]	Cognitive	MMSE	Post-intervention (end of week 12)	13 / 11	−0.459	MMSE was the only cognitive outcome reported; post-intervention values used.
Yu et al., 2021 [53]	Cognitive	ADAS-Cog	Post-intervention (end of week 24)	64 / 32	−0.203	ADAS-Cog was reported as the primary cognitive outcome; post-intervention endpoint selected.
Zuschnegg et al., 2025 [54]	Cognitive	MMSE	Post-intervention (end of week 24)	9 / 9	0.551	MMSE was the primary and only cognitive outcome reported; post-intervention assessment selected.

Note: One primary outcome per trial was selected for inclusion in the main meta-analysis to avoid unit-of-analysis errors. Outcome selection followed a prespecified hierarchy prioritizing cognitive outcomes, followed by functional or mood and behavioral outcomes when cognitive measures were not available. When multiple outcomes within the same domain were reported, global or primary outcomes and post-intervention assessments were prioritized. Effect sizes are reported as Hedges' g, with positive values indicating improvement favoring the intervention. Study-specific extraction notes summarize the rationale for outcome and time point selection.
