# Supplementary material for: Exercise Interventions for Cognitive and Functional Outcomes in Dementia: A Systematic Review and Meta-Analysis Exploring Dose Metrics, Heterogeneity, and Implementation-Relevant Factors
Source: Healthcare (Basel). 2026 Mar 9;14(5):689. doi: 10.3390/healthcare14050689 (PMC12985021; doi:10.3390/healthcare14050689)
Supplement: Supplementary file 1 [file healthcare-14-00689-s001.zip › Table S4. Risk of Bias Assessment Using the Cochrane RoB 2 Tool.pdf]

Table S4. Risk of Bias Assessment Using the Cochrane RoB 2 Tool

Study (First author, Year)	D1. Randomization process	D2. Deviations from intended interventions	D3. Missing outcome data	D4. Measurement of the outcome	D5. Selection of the reported result	Overall risk of bias
Boström et al., 2015 [33]	Some concerns	Some concerns	Low risk	Some concerns	Some concerns	Some concerns
Bracco et al., 2023 [34]	Some concerns	Some concerns	Low risk	Some concerns	Some concerns	Some concerns
Cheng et al., 2024 [35]	Some concerns	Some concerns	Low risk	Some concerns	Some concerns	Some concerns
Cox et al., 2018 [36]	Some concerns	Some concerns	Low risk	Some concerns	Some concerns	Some concerns
Fleiner et al., 2017 [37]	Some concerns	Some concerns	Low risk	Some concerns	Some concerns	Some concerns
Gebhard et al., 2022 [38]	Some concerns	Some concerns	Low risk	Some concerns	Some concerns	Some concerns
Henskens et al., 2018 [39]	Some concerns	Some concerns	Low risk	Some concerns	Some concerns	Some concerns
Krause et al., 2022 [40]	Some concerns	Some concerns	Low risk	Some concerns	Some concerns	Some concerns
Law et al., 2019 [41]	Some concerns	Some concerns	Low risk	Some concerns	Some concerns	Some concerns

Study (First author, Year)	D1. Randomization process	D2. Deviations from intended interventions	D3. Missing outcome data	D4. Measurement of the outcome	D5. Selection of the reported result	Overall risk of bias
Liu et al., 2018 [42]	Some concerns	Some concerns	Low risk	Some concerns	Some concerns	Some concerns
Morris et al., 2017 [43]	Some concerns	Some concerns	Low risk	Some concerns	Some concerns	Some concerns
Nyman et al., 2019 [44]	Some concerns	Some concerns	Low risk	Some concerns	Some concerns	Some concerns
Prick et al., 2017 [45]	Some concerns	Some concerns	Low risk	Some concerns	Some concerns	Some concerns
Sanders et al., 2020 [46]	Some concerns	Some concerns	Low risk	Some concerns	Some concerns	Some concerns
Sanprakton et al., 2025 [47]	Some concerns	Some concerns	Low risk	Some concerns	Some concerns	Some concerns
Sung et al., 2023 [48]	Some concerns	Some concerns	Low risk	Some concerns	Some concerns	Some concerns
Telenius et al., 2015 [49]	Some concerns	Some concerns	Low risk	Some concerns	Some concerns	Some concerns
Toots et al., 2017 [50]	Some concerns	Some concerns	Low risk	Some concerns	Some concerns	Some concerns
Toots et al., 2021 [51]	Some concerns	Some concerns	Low risk	Some concerns	Some concerns	Some concerns
Wu et al., 2022 [52]	Some concerns	Some concerns	Low risk	Some concerns	Some concerns	Some concerns

Study (First author, Year)	D1. Randomization process	D2. Deviations from intended interventions	D3. Missing outcome data	D4. Measurement of the outcome	D5. Selection of the reported result	Overall risk of bias
Yu et al., 2021 [53]	Some concerns	Some concerns	Low risk	Some concerns	Some concerns	Some concerns
Zuschnegg et al., 2025 [54]	Some concerns	Some concerns	Low risk	Some concerns	Some concerns	Some concerns

Note: Risk of bias was assessed using the Cochrane Risk of Bias 2 (RoB 2) tool across five standard domains. For most included trials, judgments of "some concerns" were primarily driven by limited reporting of allocation concealment procedures and the absence of blinding, which is inherent to behavioral and exercise-based interventions. In addition, prespecified statistical analysis plans were often not explicitly described, contributing to uncertainty in Domain 5. By contrast, missing outcome data were generally minimal and balanced between groups, resulting in predominantly low risk judgments for bias due to missing outcome data (Domain 3). The apparent consistency of domain-level ratings across studies reflects shared design and reporting characteristics within this literature rather than identical methodological quality across trials.
