# Supplementary material for: Exercise Interventions for Cognitive and Functional Outcomes in Dementia: A Systematic Review and Meta-Analysis Exploring Dose Metrics, Heterogeneity, and Implementation-Relevant Factors
Source: Healthcare (Basel). 2026 Mar 9;14(5):689. doi: 10.3390/healthcare14050689 (PMC12985021; doi:10.3390/healthcare14050689)
Supplement: Supplementary file 1 [file healthcare-14-00689-s001.zip › Table S5. Sensitivity and Publication Bias Analyses.pdf]

Table S5. Sensitivity and Publication Bias Analyses

Table S5. Sensitivity and publication bias analyses for the primary meta-analysis, including publication bias diagnostics, leave-one-out sensitivity analysis, and sensitivity meta-regression analyses conducted to assess robustness of the findings.

Table S5a. Publication Bias Diagnostics

**Test**	**Estimate**	**SE**	**Test statistic**	**df**	**p-value**
Egger's regression intercept	0.613	1.057	t = 0.580	20	0.568
Classic fail-safe N	12	—	—	—	—

Note: Statistical assessments of potential publication bias included visual inspection of funnel plot symmetry, Egger's regression test, and calculation of the classic fail-safe N. Results should be interpreted cautiously given the limited number of included studies.

Table S5b. Leave-One-Out Sensitivity Analysis

**Analysis condition**	**Pooled effect size (Hedges' g)**	**SE**	**95% CI**	**p-value**	**I^2 (%)**	**τ^2**
All studies included (primary analysis)	0.117	0.070	−0.021 to 0.254	0.097	51.76	0.052
Excluding Sanprakhon et al., 2025 [47]	0.106	0.046	0.015 to 0.197	0.023	~50	~0.05

Note: Sensitivity analyses were conducted to evaluate the influence of an extreme positive effect estimate (Sanprakhon et al., 2025) [47] on the pooled results. Models were re-

estimated after exclusion of this study to assess the stability of the overall findings.

Table S5c. Sensitivity Meta-Regression Analyses (Fixed-Effect Model)

**Predictor**	**β**	**SE**	**p-value**
Weeks total	−0.0049	0.0153	0.7515
Minutes per session	0.0035	0.0026	0.1362
Total Dose	0.0000	0.0001	0.8400

Note: Fixed-effect meta-regression models were conducted as sensitivity analyses to examine the robustness of associations between prespecified dose parameters and effect size estimates. Results are reported for comparison with the primary random-effects models. Model test: Qm = 2.583, df = 3, p = 0.459.
