# Supplementary material for: Exercise Interventions for Cognitive and Functional Outcomes in Dementia: A Systematic Review and Meta-Analysis Exploring Dose Metrics, Heterogeneity, and Implementation-Relevant Factors
Source: Healthcare (Basel). 2026 Mar 9;14(5):689. doi: 10.3390/healthcare14050689 (PMC12985021; doi:10.3390/healthcare14050689)
Supplement: Supplementary file 1 [file healthcare-14-00689-s001.zip › Table S6. Excluded Full-Text Studies with Reasons.pdf]

Table S6. Excluded Full-Text Studies with Reasons

Reason for exclusion	Number of studies (n)
Not randomized controlled trials or protocol-only reports	241
Ineligible population or intervention	101
Insufficient outcome data for effect size computation	50
**Total full-text articles excluded**	**392**

Note: This table summarizes full-text articles excluded after eligibility assessment, with primary reasons for exclusion recorded in accordance with the PRISMA flow diagram.
